# The abnormal level of HSP70 is related to Treg/Th17 imbalance in PCOS patients

**DOI:** 10.1186/s13048-021-00867-0

**Published:** 2021-11-15

**Authors:** Yiqing Yang, Jing Xia, Zhe Yang, Gengxiang Wu, Jing Yang

**Affiliations:** 1 Reproductive Medical Centre, Renmin Hospital of Wuhan University, Wuhan, 430060 People’s Republic of China; 2 Hubei Clinical Research Center for Assisted Reproductive Technology and Embryonic Development, Wuhan, 430060 People’s Republic of China

**Keywords:** Polycystic ovary syndrome, Heat shock protein 70, Treg, Th17, Testosterone

## Abstract

**Background:**

Polycystic ovary syndrome (PCOS) is a disease with chronic nonspecific low-grade inflammation. The imbalance of immune cells exists in PCOS. Several studies have found that heat shock protein 70 (HSP70) may be involved in the immunological pathogenesis of PCOS, but the relationship between HSP70 and Regulatory T cell (Treg)/T helper cell 17(Th17) ratio remains unclear. This study aims to explore the correlation between HSP70 and Treg/Th17 ratio and to provide evidence for the role of HSP70 in the immunological etiology of PCOS.

**Results:**

There was no significant difference in age and body mass index (BMI) between the two groups. The concentrations of basal estradiol (E_2_), basal follicle-stimulating hormone (FSH) did not show a significant difference between the two groups. The concentrations of basal luteinizing hormone (LH) (*P* < 0.01), testosterone (T) (*P* < 0.01), glucose (*P* < 0.001) and insulin (*P* < 0.001) in PCOS patients were significantly higher than those in the control group. The protein levels of HSP70 were significantly higher in serum in the PCOS group (*P* < 0.001). The percentage of Treg cells was significantly lower (*P* < 0.01), while the percentage of the Th17 cells of the PCOS group was significantly higher than that of the control group (*P* < 0.05). The ratio of Treg/Th17 in the PCOS group was significantly lower (*P* < 0.001). The concentrations of Interleukin (IL)-6, IL-17, and IL-23 were significantly higher, while the levels of IL-10 and Transforming growth factor-β (TGF-β) were significantly lower in the PCOS group (*P* < 0.001). Spearman rank correlation analysis showed a strong negative correlation of serum HSP70 levels with Treg/Th17 ratio, IL-10, and TGF-β levels. In contrast, HSP70 levels were significantly positively correlated with IL-6, IL-17, IL-23, LH, insulin, and glucose levels.

**Conclusion:**

The abnormal level of HSP70 is correlated with Treg/Th17 imbalance and corresponding cytokines, which indicates that HSP70 may play an important role in PCOS immunologic pathogenesis.

## Introduction

Polycystic ovary syndrome (PCOS) is a common reproductive endocrine disorder, affecting approximately 6–15% of women worldwide [1]. The disease is characterized by irregular menstruation, hyperandrogenemia, and polycystic ovary, with individual differences in clinical manifestations [2]. It has been reported that the risk of developing type 2 diabetes mellitus (2-DM) in PCOS women is 2–5 times higher than that in healthy women [3]. In addition, PCOS patients are at a higher risk of metabolic and cardiovascular diseases [4]. The pathogenesis of PCOS is still unclear. It is generally believed that PCOS is a disease affected by multiple factors such as heredity, endocrine, and environment. The current treatment of PCOS is still at the stage of controlling the disease progression and cannot achieve the goal of cure.

Heat shock proteins (HSPs) are a group of highly conserved protein molecules that can be produced by all prokaryotic cells and eukaryotic cells under physiological and pathological conditions including high temperature, hypoxia, virus infection, or stress [5]. HSPs participate in the regulation of cell function and play an important role in the maintenance of protein homeostasis [6]. Among these proteins, heat shock protein 70 (HSP70) is the most conserved protein, which has a variety of biological functions including molecular chaperone, regulation of immune response, anti-apoptosis, and improvement of cell tolerance to stressors [7]. Jansen observed that the expression of 5 genes encoding HSP increased in PCOS patients, of which HSP70 was the main increased protein [8]. According to several reports, the levels of HSP70 were elevated in preeclampsia [9], 2-DM [10], cancer [11], and PCOS patients [12].

Both T helper cell 17(Th17) and CD4 + CD25 + Foxp3 + regulatory T cell (Treg) belong to CD4 + T lymphocytes, but are different from Th1 and Th2 cells. Th17 cells play a key role in the pathogenesis of allergic and autoimmune diseases. They produce interleukin 17 (IL-17) and interleukin 23 (IL-23), which can recruit neutrophils and promote inflammation in infected areas. Treg cells produce anti-inflammatory cytokines such as interleukin 10 (IL-10) and transforming growth factor-β (TGF- β). Treg cells can inhibit the activity of a variety of immune cells, thus inhibit the immune response, maintain immune tolerance and keep the immune system in a balanced state. Therefore, these two types of cells inhibit each other functionally and play an opposite role in inflammation and immune response [13]. The imbalance of Treg/Th17 cells commonly exists in autoimmune diseases [14], and the balance between them plays an important role in maintaining the homeostasis of the immune system.

PCOS is considered as a pro-inflammatory state. Chronic low-grade inflammation is considered to be a key factor in the pathogenesis of PCOS [15]. It has been reported that the imbalance of T cell subsets and the abnormal cytokine concentrations exist in the ovary of women with PCOS [16]. Th1 and Th17 bias, and the decrease of Treg and Th2 cells may be involved in the pathogenesis of PCOS [17]. HSP70 plays an important role in antigen presentation, activation of macrophages and lymphocytes, as well as activation and maturation of dendritic cells [7]. Studies have shown that HSP70 has the ability to transform Th17 cells immune response mode into Treg cells immune response mode [18]. However, whether HSP70 is related to the ratio of Treg/Th17 in PCOS patients has not yet been reported.

In this work, we measured the ratio of Treg/Th17 in peripheral blood of PCOS patients, as well as the levels of HSP70 and corresponding cytokines in serum, to further explore the possible role of HSP70 in the immunological etiology of PCOS.

## Materials and methods

### Patient samples

This was a case–control study. According to previous studies, the ratios of Treg/Th17 for normal people were 2.08–3.57[19, 20], a sample size of 8–14 patients in each group was calculated with 80% power and a level of significance of 0.05. In the current study, 15 PCOS women and 15 healthy women were recruited from December 2020 to June 2021. Peripheral blood were collected. These PCOS patients were diagnosed according to the revised Rotterdam consensus [21]. The control subjects were healthy women with regular menstrual cycles and without abnormal reproduction diseases or metabolism. All these women were not treated with any medicine within 3 months. Patients with any autoimmune disease, infection within 3 months, and other possible causes of anovulation or hyperandrogenemia, 2-DM, or any cancer were excluded. Informed consent of all participants was obtained. This experiment was approved by the institutional ethics committee of the people's Hospital of Wuhan University (Ethical approval No.: WDRY2018-K027).

### Peripheral blood mononuclear cells isolation and flow cytometry analysis

Three milliliters of peripheral venous blood from PCOS and healthy women was collected respectively, and then peripheral blood mononuclear cells (PBMCs) were separated from peripheral venous blood by density gradient on Ficoll-Paque (GE Life, Sweden), which can successfully isolate the PBMCs without the need for an additional procedure to get rid of the erythrocytes/RBCs. To detect Th17 cells, lymphocytes were stimulated with the Leukocyte Activation Cocktail (BD Pharmingen, USA) for 6 h in a 37 °C humidified CO_2_ incubator. The cells were then stained with FITC-conjugated anti-CD4 (5μL/Test, BD Pharmingen, USA) in the dark at 4 °C for 30 min. Subsequently, intracellular cytokine staining was performed according to the manufacturer's protocol. The cells were fixed and permeabilized using Fix/Perm Buffer (BD Pharmingen, USA) in the dark at 4 °C for 40 min, and then were washed with Perm/Wash Buffer (BD Pharmingen, USA) before labeling the cells with PE-conjugated anti-IL-17 (20μL/Test, BD Pharmingen, USA) in the dark at 4 °C for 30 min. Similarly, to access Treg cells, lymphocytes were stained with FITC conjugated anti-CD4 (5μL/Test, BD Pharmingen, USA) and PE-CY7-conjugated anti-CD25 (5μL/Test, BD Pharmingen, USA) in the dark at 4 °C for 30 min. After fixation and permeabilization, the cells were stained intracellularly with APC-conjugated anti-Foxp3 (5μL/Test, eBioscience, USA) for 40 min. Then the lymphocytes were washed in PBS, resuspended in 200ul buffer, and then analyzed using cytoflex (American Society of Biological Sciences). Data were analyzed using FlowJo VX V10.4 software. Viable lymphocytes were gated based on forward and side scatter profiles. FITC, PE, PE -CY7 mouse IgG1, and APC rat IgG2a were used as isotype controls.

### Enzyme-linked immunosorbent assay (ELISA)

The serum concentrations of HSP70, follicle-stimulating hormone (FSH), luteinizing hormone (LH), estradiol (E_2_), testosterone (T), insulin, interleukin 6 (IL-6), IL-10, IL-17, IL-23 and TGF-β were measured by ELISA kits. All ELISA kits were purchased from Bioswamp (Wuhan, China). The ELISA was performed according to the manufacturer's instructions. The sample was diluted to 100μL (1;20), incubated with a specific capture antibody and detection antibody. All samples were detected at 450 nm optical density.

### Statistical analysis

Statistical analysis was performed using SPSS software V.19.0. The Shapiro–Wilk method was used to test whether the data were normally distributed. Comparisons between two groups were made via the unpaired two-tailed t-test. Data are shown as means ± standard. Regression assessment was made using Spearman’s rank correlation analysis. *P* < 0.05 was accepted to be statistically significant.

## Results

### Clinical and biochemical features of patients

A total of 30 patients (15PCOS women, 15 healthy women) were included in this study. Table 1 summarized the clinical characteristics of the subjects. The results showed that there was no significant difference in age and body mass index (BMI) between the two groups. The anti-Mullerian hormone (AMH) of PCOS patients was significantly higher than that of the control group (*P* < 0.01). In addition, the concentrations of basal LH (*P* < 0.01), T (*P* < 0.01), glucose (*P* < 0.001) and insulin (*P* < 0.001) in PCOS patients were significantly higher than those in the control group. Besides, there was no significant difference between the two groups in basal E2 and basal FSH.

**Table 1:** Clinical features and hormone levels of women included in this study

	Control (*n *= 15)	PCOS (*n* = 15)	*P* value
Age (year)	27.92 ± 3.40	28.75 ± 4.43	0.604
BMI (Kg/m^2^)	20.45 ± 2.04	21.58 ± 2.21	0.158
AMH (ng/ml)	3.00 ± 0.80	10.33 ± 6.59	0.001
basal FSH (mIU/ml)	7.71 ± 2.71	6.21 ± 2.78	0.309
basal LH (mIU/ml)	1.95 ± 1.82	8.92 ± 5.97	0.008
basal E_2_(pg/mL)	59.95 ± 40.01	84.82 ± 69.70	0.404
T (nmol/L)	13.98 ± 2.56	57.63 ± 52.95	0.007
Insulin (mU/L)	35.49 ± 10.19	59.69 ± 11.50	< 0.001
Glucose (mmol/L)	4.88 ± 0.79	9.32 ± 3.02	< 0.001

### Treg/Th17 ratio in the peripheral blood of PCOS patients

**Fig. 1:**
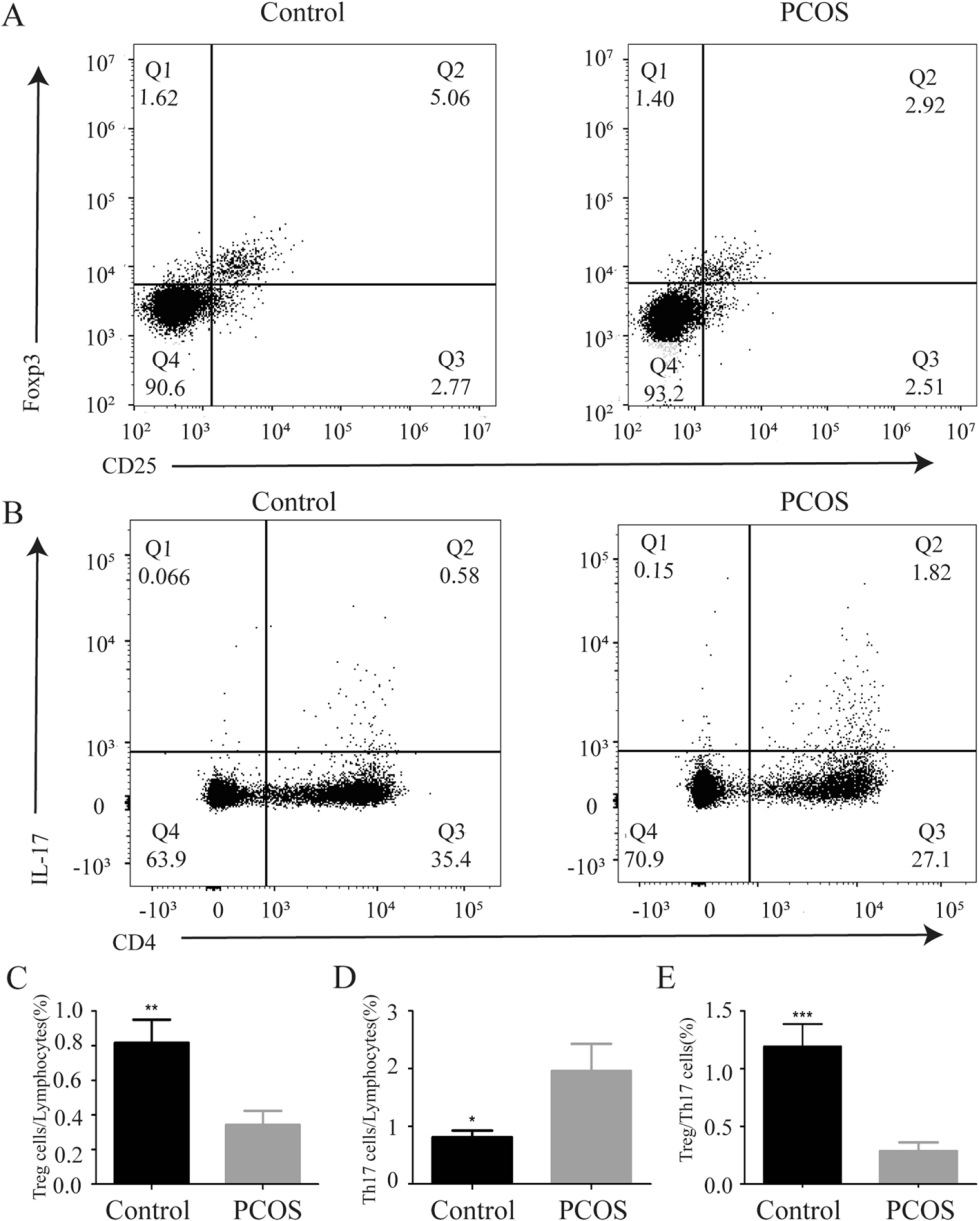
The ratio of Treg/Th17 cells in the peripheral blood of PCOS patients. **A** FCS data for the proportion of Treg cells (CD4 + CD25 + Foxp3 +) in the blood of subjects in two groups. Plot number in quadrant 2 (Q2) represents the percentage of Treg cells in CD4 + T-cells. **B** FCS data for the proportion of Th17 cells (CD4 + IL17 +) in the blood of subjects in two groups; Plot number in Q2 represents the percentage of Th17 cells in lymphocytes. **C** Bar plot of the Treg cells/lymphocytes in the blood of subjects in two groups; **D** Bar plot of the Th17 cells/lymphocytes in the blood of subjects in two groups; **E** Bar plot of the Treg/Th17 ratio in the blood of subjects in two groups. (Plot numbers represent the percentage of T-cells in the respective quadrants. Data are shown as means ± SD. ^*^P < 0.05, ^**^P < 0.01, ^***^P < 0.001)

Next, flow cytometry was used to detect the population of Th17 cells (CD4 + IL17 +) and Treg cells (CD4 + CD25 + Foxp3 +) in peripheral blood of PCOS and healthy women. As shown in Fig. 1, the Treg cells population of the PCOS group was significantly lower (*P* < 0.01) (Fig. 1A, C), while the Th17 cells population was significantly higher than that of the control group (*P* < 0.05) (Fig. 1B, D). In addition, compared with the control group, the ratio of Treg/Th17 in the PCOS group was significantly lower (*P* < 0.001) (Fig. 1E).

### HSP70 protein and cytokines levels in PCOS patients

**Fig. 2:**
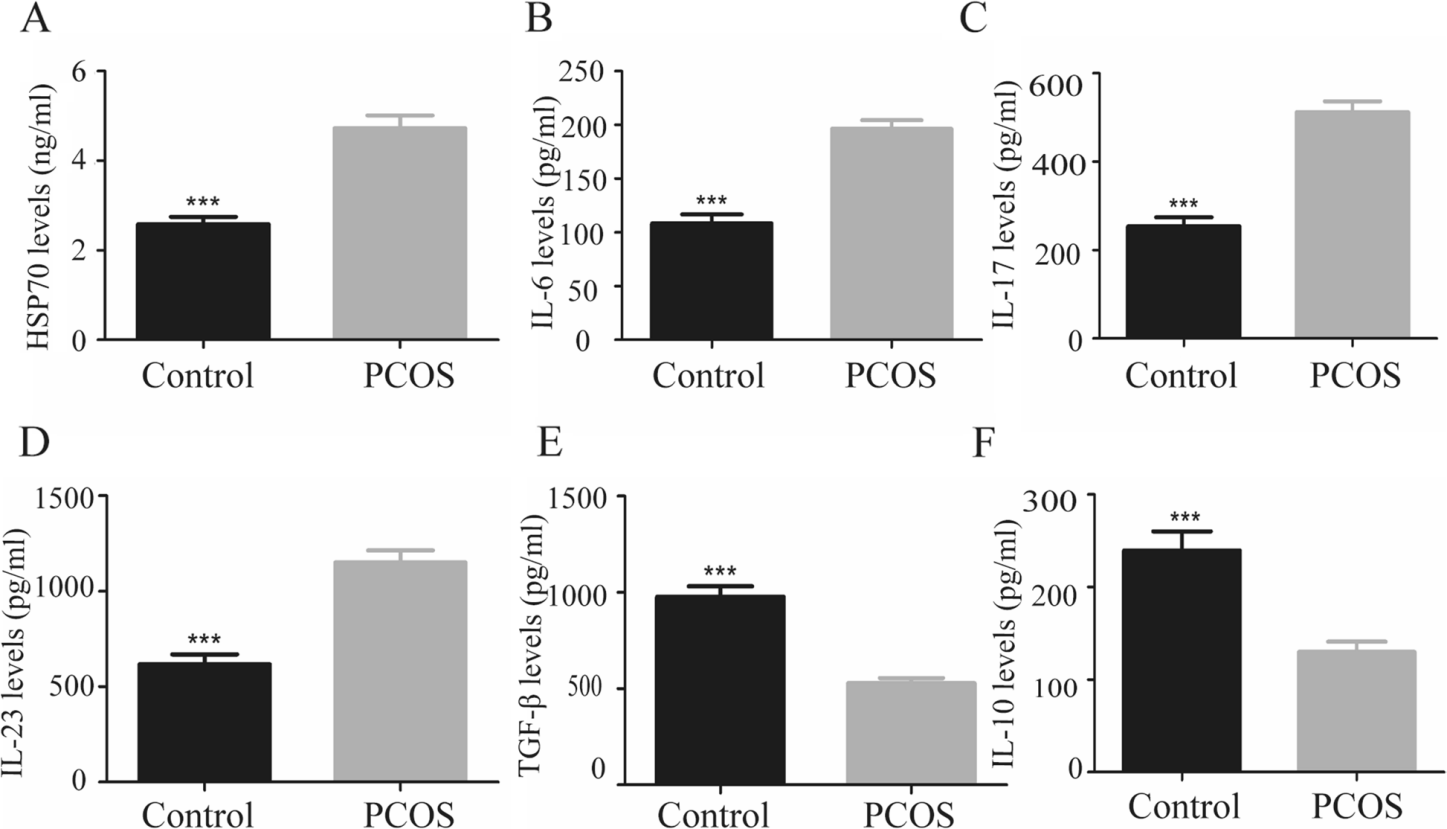
HSP70 protein and cytokines levels in PCOS and healthy women. **A** Serum HSP70 protein levels; **B** Serum IL-6 levels; **C** Serum IL-17 levels; **D** Serum IL-23 levels; **E** Serum TGF-β levels; **F** Serum IL-10 levels. (Data are shown as means ± SD. ^***^P < 0.001)

Furthermore, we detected the levels of HSP70 and cytokines corresponding to Th17 and Treg cells including IL-6, IL-10, IL-17, IL-23, and TGF- β in both groups. As demonstrated in Fig. 2A, an obvious elevation of the HSP70 level was shown in PCOS serum (*P* < 0.001). In addition, in PCOS women, the levels of IL-6 (*P* < 0.001), IL-17 (*P* < 0.001) and IL-23 (*P* < 0.001) were significantly higher (Fig. 2B, C, D), while the levels of TGF-β (*P* < 0.001) and IL-10 (*P* < 0.001) were significantly lower than those in control group (Fig. 2E, F).

### The correlation of serum HSP70 with Th17/Treg ratio, cytokines, hormones, insulin, and glucose

To explore whether HSP70 was involved in Treg/Th17 imbalance, the correlations between serum HSP70 and Treg/Th17ratio, hormones, insulin, glucose, as well as corresponding cytokines were also investigated (Table 2). Spearman rank correlation analysis showed that HSP70 levels were significantly negatively correlated with Treg/Th17 ratio (*P* < 0.05), IL-10 (*P* < 0.05) and TGF-β (*P* < 0.01) levels. In contrast, HSP70 levels were significantly positively correlated with IL-6 (*P* < 0.001), IL-17 (*P* < 0.001), IL-23 (*P* < 0.01), LH(*P* < 0.01), insulin (*P* < 0.001), and glucose (*P* < 0.01) levels. Besides, the results showed that the ratio of Treg/Th17 was significantly negatively correlated with insulin (*P* < 0.01) and glucose levels (*P* < 0.001).

**Table 2:** Spearman rank analysis for the correlation of HSP70, Th17/Treg ratio, hormones, and cytokines

		HSP70	Treg	Th17	Treg/ Th17	IL-6	IL-10	IL-17	IL-23	TGF-β	AMH	LH	T	Insulin
Treg	r	-0.294												
	*p*	0.111												
Th17	r	0.427	-0.179											
	*p*	**0.019**	0.344											
Treg/Th17	r	-0.459												
	*p*	**0.011**												
IL-6	r	0.680	-0.374	0.449	-0.526									
	*p*	** < 0.001**	**0.042**	**0.013**	**0.003**									
IL-10	r	-0.460	0.314	-0.280	0.420	-0.483								
	*p*	**0.010**	0.091	0.133	**0.021**	**0.007**								
IL-17	r	0.619	-0.540	0.545	-0.721	0.766	-0.420							
	*p*	** < 0.001**	**0.002**	**0.002**	** < 0.001**	** < 0.001**	**0.021**							
IL-23	r	0.533	-0.425	0.373	-0.538	0.762	-0.497	0.717						
	*p*	**0.002**	**0.019**	**0.043**	**0.002**	** < 0.001**	**0.005**	** < 0.001**						
TGF-β	r	-0.581	0.431	-0.251	0.440	-0.680	0.644	-0.672	-0.615					
	*p*	**0.001**	**0.017**	0.181	**0.015**	** < 0.001**	** < 0.001**	** < 0.001**	** < 0.001**					
AMH	r	0.392	-0.279	0.574	-0.551	0.721	-0.382	0.505	0.809	-0.620				
	*p*	0.119	0.277	**0.016**	**0.022**	**0.001**	0.130	**0.039**	** < 0.001**	**0.008**				
LH	r	0.739	-0.636	0.118	-0.518	0.657	-0.571	0.489	0.625	-0.607	0.406			
	*p*	**0.002**	**0.011**	0.676	**0.048**	**0.008**	**0.026**	0.064	**0.013**	**0.016**	0.244			
T	r	0.362	-0.053	0.564	-0.278	0.370	-0.117	0.230	0.234	-0.486	0.082	0.154		
	*p*	0.140	0.836	**0.015**	0.265	0.130	0.645	0.358	0.349	**0.041**	0.789	0.633		
Insulin	r	0.681	-0.532	0.323	-0.524	0.636	-0.376	0.649	0.673	-0.578	0.748	0.529	0.360	
	*p*	** < 0.001**	**0.002**	0.081	**0.003**	** < 0.001**	**0.041**	** < 0.001**	** < 0.001**	**0.001**	**0.001**	**0.043**	0.142	
Glucose	r	0.590	-0.538	0.524	-0.745	0.669	-0.346	0.724	0.639	-0.579	0.589	0.582	0.197	0.563
	*p*	**0.001**	**0.002**	**0.003**	** < 0.001**	** < 0.001**	0.061	** < 0.001**	** < 0.001**	**0.001**	**0.013**	**0.023**	0.433	**0.001**

Bold text means *P* < 0.05

## Discussion

After being stimulated by different cytokines, CD4 + T cells differentiate into different types of effector T cells including Th1, Th2, Th17, and Treg. The initial differentiation of Th17 and Treg cells share a common signaling pathway mediated by TGF-β. However, terminally differentiated cells perform the opposite function. Th17 cells can lead to autoimmune response and inflammation, while Treg cells inhibit these inflammatory phenomena and maintain immune homeostasis [22]. Th17 and Treg cells also maintain the balance of maternal–fetal interface immunity and play an important role in recurrent pregnancy loss [23] and preeclampsia [24]. In addition to genetic and environmental factors, the important role of the immune system in PCOS has received widespread attention in recent years.

In our study, the results of flow cytometry showed that compared with the control group, the proportion of Treg cells in PCOS patients decreased, while the proportion of Th17 cells increased, and the proportion of Treg/Th17 cells in peripheral blood of PCOS patients decreased significantly. A series of previous studies have shown that compared with healthy women, the number of activated T cells in ovarian follicular fluid of PCOS patients increased [25], while the number of Treg cells in peripheral blood of PCOS patients decreased [26]. The imbalance of Th1/Th2 and Th17/Treg was also observed in PCOS patients. The percentage of CD4 + CD25 + Foxp3 + T cells in PCOS patients decreased, and the proportion of Th17 subsets increased, although the difference was not statistically significant [17]. These results were consistent with ours. It has been reported that neonatal exposure of female rats to androgens can increase the relative and absolute number of CD4 + CD25 + Foxp3 + Treg cells in peripheral blood [27]. Androgen can preserve the number of male Treg cells directly or indirectly through its metabolites [28]. Our results showed that the androgen levels of PCOS patients were higher, but the ratio of Treg cells was lower than that of the control group. Spearman correlation analysis demonstrated that there was no correlation between androgen levels and Treg cells. However, a significant positive correlation was observed between the ratio of Treg cells and LH levels. The reason may be that the serum hormones in PCOS patients, except LH, other hormones such as FSH, E_2_, and T, will not affect the ratio of Treg cells. Congenital disability of peripheral Tregs may exist in PCOS women. According to a previous study, the decrease of Tregs in peripheral blood of PCOS patients was due to the inherent low responsiveness of the body to IL-2, which led to the abnormal activation of signal transducer and activator of transcription 5B and the reduction of Foxp3 expression [26].

In our previous study, elevated levels of inflammatory molecules in peripheral blood of PCOS rats were observed, including C-reactive protein, IL-6, and tumor necrosis factor-α [29]. In this study, we detected the levels of Treg and Th17 cell-related cytokines including TGF-β, IL-10, IL-6, IL-17, and IL-23 in the serum of PCOS patients. As expected, we found that Treg cell-related cytokines TGF-β and IL-10 decreased in PCOS patients, while the levels of Th17 cell-related cytokines IL-6, IL-17, and IL-23 increased. This result further confirms the view that chronic low-grade inflammation is involved in the pathogenesis of PCOS. Besides, we found that the levels of insulin and glucose were negatively correlated with the proportion of Treg cells and Treg/Th17 ratio. Insulin resistance (IR) may be one of the main factors in the development of PCOS. IR and hyperinsulinemia play an important role in the pathophysiology of PCOS. They aggravate the disorder of the reproductive endocrine and metabolism of glucose and lipid in PCOS patients [30]. IR is one of the key factors affecting the efficacy of PCOS treatment. In recent years, more and more evidence showed that inflammation played an important role in IR. IR is related to immune factors such as adipocytokines and leptin. Chronic subclinical inflammation may be the initial cause of IR [31]. Relevant studies have shown that IR can lead to inflammatory response and Th17/Treg imbalance, which can be rescued by IL-6[32].

As chaperone proteins, HSPs participate in the assembly of proteins in cells and contribute to protein homeostasis [6]. HSP70 has attracted more and more attention due to its important function in gametogenesis or pregnancy regulation [33]. In this study, ELISA results showed that the serum HSP70 levels of PCOS patients were increased, which was consistent with the previous research [34]. The increase of serum HSP70 levels was related to IR, oxidative stress, and low-grade chronic inflammation in PCOS individuals. Elevated serum HSP70 levels were considered to indicate the ovarian damage of transgenic mice under oxidative/ischemic stress [35].

In addition to the chaperone function, HSP70 can also stimulate and inhibit inflammation. The correlation between HSP70 and Treg/Th17 cells ratio was studied for the first time in our work. Spearman rank correlation analysis demonstrated that the levels of HSP70 in human serum were significantly negatively correlated with Treg/Th17 ratio. The ability of HSP70 to induce autoimmune response may be part of natural autoimmunity, or it may be related to pathological autoimmune diseases. Anti-HSP70 antibody has been confirmed in the cord and may play a role in the normal immune system [36].

HSPs are stress-induced proteins with immunomodulatory properties. They contain a peptide binding domain, which can bind proteins and non-protein molecules with exposed hydrophobic residues. The function of antigen presentation and the ability to induce cytokines in vitro are the results of the binding between HSPs and molecules or molecular chaperone role of HSPs [37]. In inflammatory disease models, T cells that respond to heat shock proteins inhibit the disease by producing anti-inflammatory cytokines. The anti-inflammatory activity of HSP-specific T cells depends on their recognition of endogenous HSP epitopes. It has been reported that these T cells can be induced by the conservative sequence of HSP of microorganisms. The upregulation of endogenous HSP expression induced by drugs can promote the production of anti-inflammatory T cells [38]. HSP70 can induce protective, anti-inflammatory regulatory T-cell response [36].

In our study, serum HSP70 levels were significantly negatively correlated with the Treg/Th17 ratio. The possible reason may be that in addition to regulating protein homeostasis, HSPs also participate in the enhancement of immune response under a variety of stress conditions, including fever, oxidative stress, and inflammatory cytokine signaling activation during viral infection. In response to these stress signals, HSPs mediate the constitutive and inducible danger signals that activate the immune response [39]. HSP70 has been reported to activate the innate immune system [40]. Studies have shown that the E3 ligase Stub1, which is expressed in response to danger signals during inflammation, is responsible for the ubiquitination of Foxp3 with the help of HSP70 chaperone protein, which leads to the degradation of major Treg cell transcription factors [39]. The high level of HSP70 directly promotes the expression of the Th17 gene after T cell receptor stimulation. This effect stems from the direct intracellular interaction of HSP70 with a RISC complex. The activity of HSP70 in Th17 promotion depends on the regulation of a set of specific miRNA expressions. Selective inhibition of these microRNAs or directly blocking the function of HSP70 will downregulate the expression of the Th17 gene [41].

A previously conducted study showed that heat shock proteins interact with T cells mainly through Toll-like receptors 2(TLR2) and Toll-like receptors 4(TLR4) [42]. It has also been found that increased cytokine synthesis and endometrial inflammation in PCOS patients are associated with androgen-induced TLR4/IRF-7/NF-κB signaling [43]. TLR4 activates and induces recruitment of downstream proteins, such as myeloid differentiation primary response protein 88 [44], resulting in the secretion of pro-inflammatory cytokines including IL-6 and tumor necrosis factor-α [45]. Another research observed that both in vitro and in vivo, exosomes derived from heat-stressed tumour cells, which contain abundant HSP70, were more effective at stimulating dendritic cells to secrete IL-6 for converting Tregs into Th17 cells. The antitumour effects of both exosomes originating from tumours and heat-stressed tumour cells were found to be dependent upon IL-6 [46]. Here, we speculate that in PCOS patients, increased level of HSP70 enhances the release of pro-inflammatory cytokines such as IL-6 via the TLR4-NFkB pathway, leading to an imbalance of Treg and Th17 cells. However, the exact mechanism still needs to be further explored. In our study, spearman rank correlation analysis showed that serum HSP70 levels were significantly negatively correlated with IL-10 and TGF-β levels, while significantly positively correlated with IL-6, IL-17, IL-23, and other inflammatory cytokines. Our previous study has shown that the levels of serum HSP70in PCOS rats were decreased, and they were strongly negatively correlated to T, LH, and inflammatory factors such as C-reactive protein, IL-6, IL-18, and tumor necrosis factor-α [29]. This is contrary to our results in PCOS patients. The possible reason is that the transient hyperphysiological T level caused by the construction of the PCOS rat model down-regulated the level of HSP70 in rat serum. HSP72 is another member of the HSP70 families. According to the previous research, transient hyperphysiological T level can down-regulate the expression of HSP72. The regulation of HSP70 expression is mediated by heat shock transcription factor (HSF)-1. T can induce the inhibition of HSP72 expression or HSF1 activation, which may directly block the trimerization and phosphorylation of HSF1, or indirectly inhibit HSF1 activation [47], leading to the reduced level of HSP70.

Insulin has the capacity to promote the occurrence and development of PCOS through PI3K and MAPK signaling pathways [48]. Here, our current investigation found that HSP70 was significantly positively correlated with insulin and glucose level. Several studies have demonstrated that serum HSP70 levels were elevated in patients with type 1 or type 2 diabetes [49, 50]. Furthermore, serum HSP70 concentrations were found to be significantly higher in women with gestational diabetes than in healthy pregnant women [51]. In contrast, recent research observed that DNAJB3(a member of the HSP40 family) co-chaperone was downregulated in obese and diabetic patients. Failure of the heat shock response is a key event leading to IR and 2-DM [52]. The reason may be that though intracellular HSP70 can counteract IR [53], extracellular HSP70 has been proved to be associated with IR in vivo and to have the capacity to cause pancreatic β-cell dysfunction in vitro [54].

Our research also proved that insulin levels and AMH levels were significantly positively correlated. The relationship between insulin levels and AMH levels in PCOS patients is controversial. A series of studies have found that compared with PCOS patients without IR, the concentration of AMH in PCOS patients with IR is significantly higher [55]. The serum AMH is positively correlated with Homeostasis model assessment (HOMA)-IR [56], which is consistent with our results. However, other studies have not found a link between AMH and IR [57, 58]. It has also been reported that there is a negative correlation between AMH and HOMA-IR [59]. These contradictory data may be partly caused by the heterogeneity of the study population.

Our study has several limitations. Due to its cross-sectional design, the causal relationship between HSP70 and Treg/Th17 is unclear. In addition, due to the limitation of sample collection, the small number of patients limits the statistical power and the generalizability of our findings. Besides, both insulin and glucose are not tested under fasting conditions, thus the judgment of IR is not clear. The relationship between HSP70 and Treg/Th17 ratio still needs further research. Although this study confirmed that the upregulation of HSP70 levels was negatively correlated with Treg/Th17 ratio, it is still necessary to study the specific regulatory mechanism of molecular biology to provide new targets for disease diagnosis and treatment.

## Conclusion

Herein, the imbalance of Treg/Th17 exists in peripheral blood of PCOS patients, and the abnormally elevated HSP70 in serum of PCOS patients is related to Treg/Th17 imbalance and cytokines, suggesting that HSP70 may play an important role in the immunological etiology of PCOS. Specific mechanisms and causal relationships between HSP70 and Treg/Th17 ratio still need further study.

## Data Availability

The datasets used and/or analysed during the current study are available from the corresponding author on reasonable request.

## Abbreviations

PCOS: Polycystic ovary syndrome
HSPs: Heat shock proteins
HSP70: Heat shock protein 70
IR: Insulin resistance
BMI: Body mass index
FSH: Follicle-stimulating hormone
E_2_: Estradiol
LH: Luteinizing hormone
T: Testosterone
AMH: Anti-Mullerian hormone
IL-6: Interleukin 6
IL-10: Interleukin 10
IL-17: Interleukin 17
IL-23: Interleukin 23
TGF-β: Transforming growth factor-β
Th17: T helper cell 17
Treg: Regulatory T cell
2-DM: Type 2 diabetes mellitus
TLR2: Toll-like receptors 2
TLR4: Toll-like receptors 4
HOMA: Homeostasis model assessment
HSF: Heat shock transcription factor
